# Postoperative Density of Residual Hematoma Is a Predictor of Chronic Subdural Hematoma Recurrence—A Single-Center Retrospective Study

**DOI:** 10.3390/neurolint18080157

**Published:** 2026-08-20

**Authors:** Faisal Sawab, Tim Lampmann, Haitham Alenezi, Lars Eichhorn, Mohammed Banat, Hartmut Vatter, Marcus Thudium, Motaz Hamed

**Affiliations:** 1 Department of Neurosurgery, University Hospital Bonn, 1, 53127 Bonn, Germanymohammed.banat@ukbonn.de (M.B.); hartmut.vatter@ukbonn.de (H.V.); motaz.hamed@ukbonn.de (M.H.); 2 Department of Anesthesiology, Intensive Care Medicine and Pain Medicine, Helios Hospital Krefeld, Krefeld 47805, Germany; lars.eichhorn@helios-gesundheit.de; 3 Department of Anaesthesiology and Intensive Care Medicine, University Hospital Bonn, 53127 Bonn, Germany; marcus.thudium@ukbonn.de

**Keywords:** cSDH, burr-hole, trepanation, control

## Abstract

**Background/Objectives**: Chronic subdural hematoma (cSDH) is one of the most common neurosurgical disorders in older adults and remains associated with a relevant recurrence rate despite standardized surgical treatment. Identifying postoperative factors that predict recurrence may improve postoperative surveillance and surgical strategy. We therefore investigated whether the density of the residual hematoma on early postoperative computed tomography (CT) is associated with recurrence after burr-hole trepanation. **Methods**: We retrospectively analyzed all consecutive patients who underwent burr-hole trepanation for cSDH at our institution between 2016 and 2021. Residual hematoma density was measured in Hounsfield units (HU) on postoperative CT scans. Recurrence was defined as symptomatic hematoma reaccumulation requiring reoperation. **Results**: A total of 223 patients met the inclusion criteria. The overall recurrence rate was 20.2%. Mean residual hematoma density was significantly higher in the recurrence group than in the non-recurrence group (23.2 ± 13.1 HU vs. 19.0 ± 9.1 HU; *p* = 0.005). ROC analysis identified an optimal cutoff value of 19 HU (AUC 0.578, 95% CI 0.511–0.645; *p* = 0.022) with sensitivity of 57.8%, and a specificity of 57.9%. Patients with residual hematoma density ≥19 HU had a significantly higher recurrence rate than those with lower density (25.7% vs. 15.6%; *p* = 0.043). In multivariable logistic regression, higher residual density remained an independent predictor of cSDH recurrence (*p* = 0.008, OR 1.9; 95% CI 1.2–3.0). **Conclusions**: Higher postoperative residual hematoma density is an independent predictor of recurrence after burr-hole trepanation for cSDH. Residual density on early postoperative CT may serve as a simple and clinically accessible imaging marker of incomplete hematoma clearance and may help refine postoperative risk assessment. Nevertheless, the low AUC limits interpretation of predictive performance.

## 1. Introduction

Chronic subdural hematoma (cSDH) is a frequent and increasingly important neurosurgical condition, particularly in the elderly population, and its incidence is expected to rise further with population ageing and the growing use of antithrombotic therapy [1,2].

It is typically characterized by the accumulation of blood products in the subdural space after minor trauma, brain atrophy, or coagulation impairment, and its diagnosis is generally established by cranial computed tomography (CT), which allows reliable assessment of hematoma size, density, and mass effect [3,4,5].

Although burr-hole trepanation with closed-system drainage remains the standard surgical treatment because of its favorable balance of efficacy, safety, and simplicity, recurrence remains a major limitation of care [5,6,7,8].

Recurrence is clinically relevant because it may necessitate repeat surgery, prolong hospitalization, delay rehabilitation, and complicate the management of anticoagulant or antiplatelet therapy [9].

Despite extensive research, there is still no universally accepted strategy that reliably identifies all patients at risk of recurrence before or immediately after surgery [10,11,12,13,14].

Recent studies and reviews have increasingly emphasized the value of radiological predictors, including hematoma density, internal architecture, postoperative residual volume, and postoperative pneumocephalus, as markers of recurrence risk [15,16,17,18,19].

Among these variables, residual hematoma density on postoperative CT has not been explored as extensively as residual volume or postoperative air [16,17,18].

A denser residual collection may reflect incomplete evacuation, persistent coagulated material, or a more organized postoperative cavity environment that is less likely to resolve spontaneously.

We therefore hypothesized that higher residual hematoma density after burr-hole trepanation is associated with a greater risk of recurrence.

The aim of the present study was to evaluate the association between postoperative residual hematoma density and recurrence in patients surgically treated for cSDH, and to determine whether this simple postoperative imaging marker has independent prognostic value. Moreover, if residual hematoma density is confirmed as a prognostic marker, we want to establish an easy-to-use cutoff for routine use.

## 2. Materials and Methods

### 2.1. Study Design

We conducted a retrospective single-center cohort study including all consecutive patients who underwent burr-hole trepanation for cSDH at our institution between 2016 and 2021. Reporting followed the STROBE standard. Surgery was performed in symptomatic patients, in cases with space-occupying hematoma, or when antithrombotic therapy could not be safely interrupted for an extended period.

Patients were excluded if postoperative CT imaging was unavailable, follow-up was insufficient for definitive outcome assessment, or acute postoperative rebleeding was present. Baseline demographic, clinical, and perioperative data were retrieved from a computerized institutional database.

### 2.2. Clinical Management

Perioperative management of antithrombotic therapy followed institutional practice and depended on the clinical urgency of surgery. In urgent cases, anticoagulation or antiplatelet effects were reversed preoperatively whenever possible. In elective cases, antithrombotic agents were discontinued according to drug class and clinical judgment.

Placement of subdural drainage was left to the discretion of the attending neurosurgeon. Postoperative CT scans were usually obtained within 3 days to assess for complications and to guide drain removal. Patients were discharged home or to geriatric rehabilitation depending on their clinical condition. Follow-up was performed 3 to 6 weeks after surgery in the outpatient clinic and included clinical assessment and radiological imaging when indicated. Recurrence was defined as symptomatic hematoma reaccumulation requiring reoperation.

### 2.3. Calculation of Density

Postoperative CT scans were performed with the same scanner (Philips IQon Spectral CT) and analyzed using iPlan Smartbrush software (Version 5.0.1.2, Brainlab, Feldkirchen, Bavaria, Germany). Residual hematoma density was measured in Hounsfield units (HU) on postoperative CT images. The measurement region was restricted to the residual hematoma between the brain surface and the skull, and drainage devices and obvious artifacts were excluded. Mean density values were calculated for each cavity (bilateral hematomas were counted as two individual cases).

### 2.4. Statistical Analysis

Statistical analyses were performed using SPSS Statistics version 29. Continuous variables were assessed for normality and compared using the unpaired *t*-test or Mann–Whitney U test, as appropriate. Categorical variables were compared using the chi-square test or Fisher’s exact test. A *p*-value < 0.05 was considered statistically significant.

Receiver operating characteristic (ROC) analysis was performed to evaluate the predictive value of residual hematoma density for recurrence. The optimal cutoff was determined using the Youden index. Sensitivity, specificity, area under the curve (AUC), and 95% confidence intervals were calculated. Multivariable logistic regression analysis was performed to determine whether residual density remained an independent predictor of recurrence.

## 3. Results

A total of 223 patients fulfilled the inclusion criteria and were included in the final analysis. The overall recurrence rate was 20.2% (45/223). Baseline characteristics of the study population are summarized in Table 1.

Mean residual hematoma density was significantly higher in the recurrence group than in the non-recurrence group (23.2 ± 13.1 HU vs. 19.0 ± 9.1 HU; *p* = 0.005).

**Figure 1:**
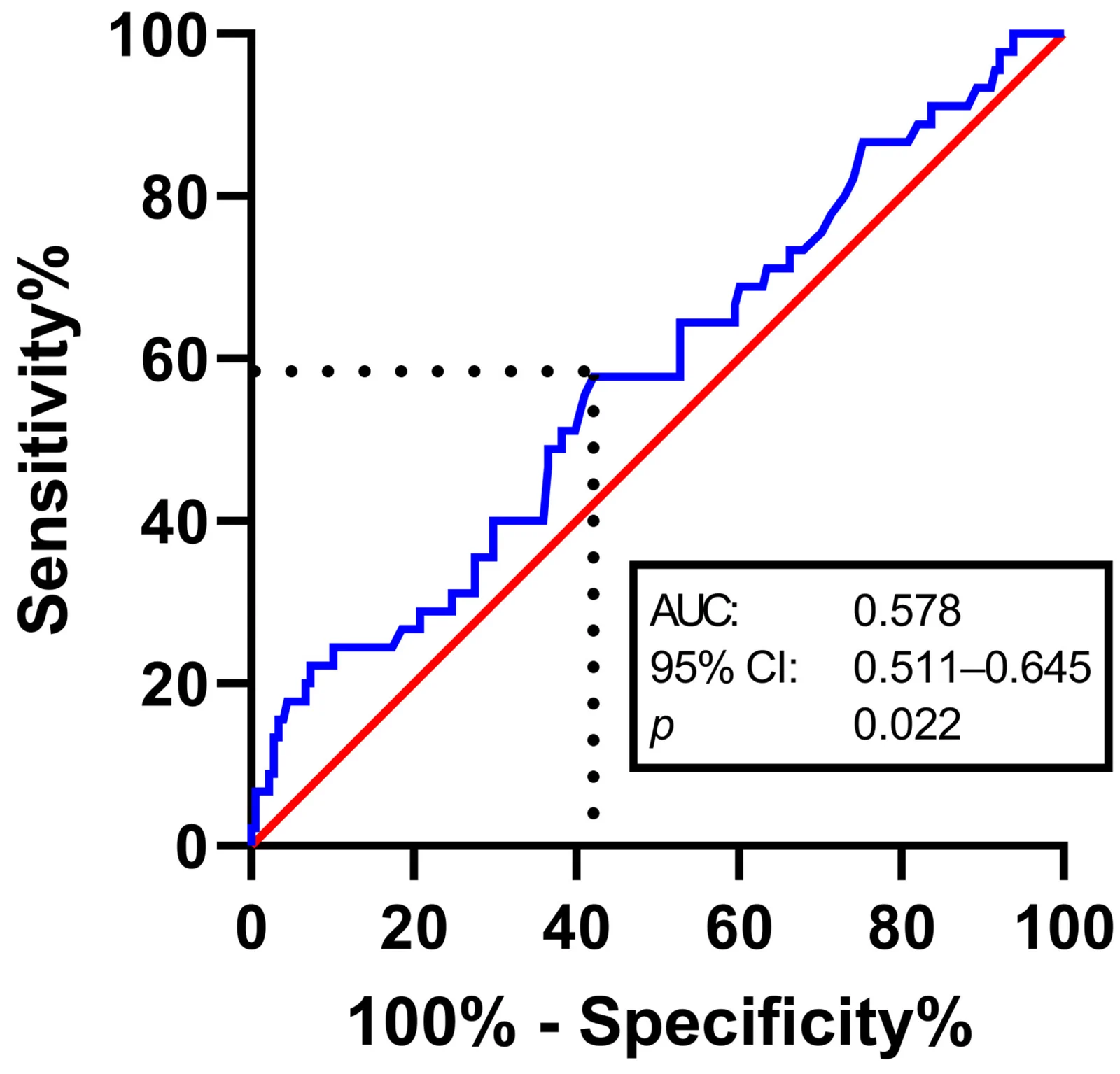
Receiver-operating characteristic curve illustrating density of residual hematoma in prediction of recurrence of chronic subdural hematoma.

ROC analysis identified an optimal cutoff value of 19 HU for recurrence prediction. However, the overall discriminative performance was modest, with an AUC of 0.578 (95% CI 0.511–0.645; *p* = 0.022), a sensitivity of 57.8%, and a specificity of 57.9% (Youden’s J = 0.157) (Figure 1).

When patients were dichotomized according to this cutoff, baseline characteristics did not differ significantly between the low-density and high-density groups. Age, sex, hematoma laterality, antiplatelet use, direct oral anticoagulant use, duration of hospitalization, number of drainages, and duration of surgery were comparable between groups (Table 2).

The only significant difference between the groups was the recurrence rate, which was higher in the high-density group than in the low-density group (25.7% vs. 15.6%; *p* = 0.043). Residual hematoma density >19 HU was associated with an odds ratio for recurrence of 1.88 (95% CI 0.97–3.65).

**Table 2:** Comparison of different densities dichotomized with a cut-off of 19 HU in patients treated for CSDH.

	Low Density	High Density	*p*-Value
**Overall**	122 (100%)	101 (100%)	
**Median Age [years] (IQR)**	80 (74–86)	79 (73–84.5)	0.424
**Sex**			0.663
Female	36 (29.5%)	33 (32.7%)	
Male	86 (70.5%)	68 (67.3%)	
**Side**			0.303
Right	36 (29.5%)	36 (35.6%)	
Left	43 (35.2%)	39 (38.6%)	
Bilateral	43 (35.2%)	26 (25.7%)	
**Platelet Aggregation Inhibitor**	52 (42.6%)	39 (38.6%)	0.585
**Direct Oral Anticoagulants**	31 (25.4%)	28 (27.7%)	0.870
**Duration of Hospitalisation [days] (IQR)**	7 (4–11)	5 (4–8.5)	0.165
**Number of Drainages per cavity**			0.975
0	7 (5.7%)	6 (5.9%)	
1	61 (50.0%)	49 (48.5%)	
2	54 (44.3%)	46 (45.5%)	
**Duration of Surgery [min] (IQR)**	33.5 (24–47)	34.5 (25–45)	0.920
**Recurrence**	19 (15.6%)	26 (25.7%)	**0.043**

IQR: Interquartile Range. Significant *p*-values < 0.05 are marked in bold.

In multivariable logistic regression, higher residual density remained an independent predictor of cSDH recurrence (*p* = 0.008, OR 1.9; 95% CI 1.2–3.0; Nagelkerke’s R^2^ = 25%).

## 4. Discussion

Chronic subdural hematoma remains one of the most common neurosurgical diseases in older adults, and recurrence after surgery continues to be a major practical and scientific challenge. In the present study, higher postoperative residual hematoma density was independently associated with recurrence after burr-hole trepanation. This finding is important because it suggests that a simple, routinely available postoperative CT parameter may provide prognostic information that goes beyond conventional clinical assessment [20].

Our results are consistent with the current view of cSDH as a dynamic and biologically active disease rather than a static blood collection. cSDH develops through a combination of membrane formation, inflammatory activation, fragile neovascularization, and repeated microhemorrhage, all of which contribute to hematoma persistence and recurrence [17,19,21,22]. In this setting, the radiological appearance of the hematoma may reflect the underlying organization and biological activity of the lesion rather than serving only as a descriptive feature. A higher residual density after surgery may therefore indicate persistent coagulated or organized material within the subdural cavity, suggesting incomplete clearance and a greater likelihood of reaccumulation. This interpretation is supported by earlier work on radiological predictors of recurrence.

A systematic review and meta-analysis demonstrated that hyperdense hematoma components, laminar and separated architecture, larger thickness, and greater midline shift were associated with recurrence after surgery [7].

Likewise, recurrence score studies have incorporated density as a relevant predictive variable, and a clinical risk score developed by Stanišić and Pripp identified postoperative hematoma density and volume as independent risk factors for recurrence [10]. More recent risk-stratification work by Lioi et al. also found hematoma density to be a meaningful component of recurrence prediction [23]. Our findings extend these observations to the postoperative setting and indicate that residual density after evacuation may serve as a practical marker of remaining recurrence risk.

The biological plausibility of this association is strong. A denser residual collection may contain a greater proportion of fibrin-rich, organized, or partially clotted hematoma that is not readily removed by standard irrigation. Such residual material may remain biologically active, perpetuating inflammatory signaling, membrane activity, and microvascular leakage within the postoperative cavity [21]. It may also be associated with less complete brain re-expansion, which is a key factor for postoperative cavity stability and a recognized contributor to recurrence. From this perspective, residual density can be understood as a surrogate marker of incomplete evacuation and an unfavorable postoperative microenvironment.

Our study also fits well with the growing literature on postoperative cavity conditions, especially pneumocephalus. A 2025 retrospective cohort analysis from our own group showed that postoperative pneumocephalus was associated with recurrence and prolonged hospitalization, and a 2025 systematic review and meta-analysis confirmed a strong association between postoperative pneumocephalus and recurrence [18]. These findings reinforce the notion that recurrence depends not only on preoperative hematoma characteristics but also on the postoperative cavity state. Residual clot, postoperative air, and incomplete cavity collapse likely act together to maintain a space that favors reaccumulation.

Within that framework, residual density adds another layer of information by identifying patients in whom the postoperative cavity still contains a biologically relevant amount of hematoma material. The clinical value of residual density lies in its simplicity. Unlike radiomics-based approaches or labor-intensive volumetric analyses, density measurement is immediately available on routine postoperative CT and can be applied in everyday clinical workflows [15,16]. This makes it potentially useful for postoperative risk stratification, especially in older patients for whom repeated imaging and hospital visits may be burdensome.

While recent trials evaluated pharmacological approaches such as dexamethasone, which showed no improvement in functional outcome, subsequent embolization of the middle meningeal artery (MMAE) has emerged as a new approach to reducing recurrence. [24,25,26,27] However, this once again constitutes an invasive procedure with associated procedural risks. The advantage of radiological assessment of residual hematoma density lies in its prognostic value even before repeat intervention and, if necessary, in helping to determine whether further steps are needed. So, a parameter that is easy to obtain but still independently informative is particularly attractive in cSDH, where recurrence prediction is most useful when it can influence surveillance intensity, discharge planning, and the threshold for reimaging.

Our ROC analysis showed only modest discriminative performance, which should temper overinterpretation. The 19 HU cutoff identified in our cohort should be regarded as cohort-specific and hypothesis-generating rather than universally applicable. Still, the fact that residual density remained an independent predictor in multivariable analysis indicates that it contains information not fully captured by age, sex, antithrombotic medication, hematoma laterality, drainage use, or operative duration.

In that sense, the parameter appears to reflect a distinct aspect of postoperative cavity behavior. The result also raises a practical surgical question. If higher residual density reflects retained organized clot or insufficient washout, then more thorough intraoperative irrigation might reduce the amount of residual material and potentially lower recurrence risk. This is a plausible hypothesis, but not yet a proven recommendation. Nevertheless, it emphasizes that the surgeon’s endpoint should extend beyond decompression alone and include the quality of postoperative cavity clearance [28,29,30,31].

Our findings are also consistent with the broader movement toward structured recurrence prediction in cSDH. Recent scoring systems have shown that recurrence risk can be meaningfully stratified by integrating radiological variables such as density, architecture, cortical atrophy, and postoperative cavity features [10,15,23]. This is clinically relevant because postoperative management after cSDH is often individualized, and a reproducible imaging marker could help identify patients who warrant closer surveillance or lower thresholds for re-evaluation [5,6,7,32,33]. Although no single marker will fully solve recurrence prediction, a parameter as accessible as residual density may contribute meaningfully to a broader risk model.

The study has several limitations. First, it was retrospective and single-center, which limits generalizability and leaves room for unmeasured confounding. Second, postoperative CT timing was not perfectly standardized, and density values may vary depending on the interval between surgery and imaging. Third, recurrence was defined by reoperation, which is a clinically robust endpoint but may underestimate smaller recurrent collections managed conservatively. Finally, due to the low AUC of 0.578, the discriminative ability of the ROC analysis is limited and should be interpreted with caution. Therefore, the cutoff identified in this cohort requires external validation before it can be incorporated into clinical decision-making.

Despite these limitations, the study has important strengths. It includes a consecutive real-world cohort, uses a clinically meaningful recurrence endpoint, and evaluates a parameter that is easy to obtain in routine practice. The association between postoperative residual density and recurrence remained evident in both univariable and multivariable analyses, strengthening the credibility of the finding. For a disease in which recurrence remains common despite standardized surgery, even a modest but reproducible imaging marker may help refine follow-up pathways and improve individualized care.

## 5. Conclusions

Higher postoperative residual hematoma density was an independent predictor of recurrence after burr-hole trepanation for chronic subdural hematoma.

Residual density on early postoperative CT may therefore serve as a simple, reproducible imaging marker of incomplete hematoma clearance and postoperative risk.

## Figures and Tables

**Table 1:** Baseline characteristics.

**Overall**	223 (100%)
**Median Age [years] (IQR)**	79 (74–85)
**Sex**	
Female	69 (30.9%)
Male	154 (69.1%)
**Side**	
Right	72 (32.3%)
Left	82 (36.8%)
Bilateral	69 (30.9%)
**Platelet Aggregation Inhibitor**	91 (40.8%)
**Direct Oral Anticoagulants**	59 (26.5%)
**Midline Shift**	163 (73.1%)
**Duration of Hospitalisation [days] (IQR)**	6 (4–10)
**Number of Drainages per cavity**	
0	13 (5.8%)
1	110 (49.4%)
2	100 (44.8%)
**Duration of Surgery [min] (IQR)**	34 (25–45)
**Recurrence**	45 (20.2%)

IQR: Interquartile Range.

## Data Availability

All data generated or analyzed during this study are included in this published article.
